# Over-expression and characterization of NS3 and NS5A of Hepatitis C virus genotype 3a

**DOI:** 10.1186/1475-2859-12-111

**Published:** 2013-11-16

**Authors:** Muhammad Ikram Anwar, Mazhar Iqbal, Mohammad S Yousef, Moazur Rahman

**Affiliations:** 1 Drug Discovery and Structural Biology group, Health Biotechnology Division, National Institute for Biotechnology and Genetic Engineering (NIBGE), Faisalabad, Pakistan; 2 Department of Physics, College of Arts and Sciences, Southern Illinois University, Edwardsville, Edwardsville, IL 62026-1654, USA; 3 Biophysics Department, Faculty of Science, Cairo University, Giza 12613, Egypt

**Keywords:** HCV, NS3, NS5A, Genotype 3a, Protease, Helicase, Viral replication, Protein expression, CD spectroscopy, FT-IR

## Abstract

**Background:**

Hepatitis C virus (HCV) is a common and leading cause for liver cirrhosis and hepatocellular carcinoma. Current therapies to treat HCV infection are shown to be partially effective and poorly tolerated. Therefore, ample efforts are underway to rationally design therapies targeting the HCV non-structural proteins. Most of the work carried out in this direction has been focusing mainly on HCV genotype 1. Two direct-acting antiviral agents (DAAs) Telaprevir and Boceprevir are being used against genotype 1a infection in combination therapy with interferon and ribavirin. Unfortunately these DAAs are not effective against genotype 3a. Considering the wide spread infection by HCV genotype 3a in developing countries especially South Asia, we have focused on the recombinant production of antiviral drug targets NS3 and NS5A from HCV genotype 3a. These protein targets are to be used for screening of inhibitors.

**Results:**

High-level expression of NS3 and NS5A was achieved at 25°C, using ~1 and 0.5 mM Isopropyl β-D-1-thiogalactopyranoside (IPTG), respectively. Yields of the purified NS3 and NS5A were 4 and 1 mg per liter culture volume, respectively. Although similar amounts of purified NS3 were obtained at 25 and 14°C, specificity constant (*K*_cat_/*K*_m_) was somewhat higher at expression temperature of 25°C. Circular dichroism (CD) and Fourier-transform infrared (FT-IR) spectroscopy revealed that both NS3 and NS5A contain a mixture of alpha-helix and beta-sheet secondary structures. For NS3 protein, percentages of secondary structures were similar to the values predicted from homology modeling.

**Conclusions:**

NS3 and NS5A were over-expressed and using Nickel-affinity method both proteins were purified to ~ 95% purity. Yield of the purified NS3 obtained is four fold higher than previous reports. CD spectroscopy revealed that difference in activity of NS3 expressed at various temperatures is not related to changes in global structural features of the protein. Moreover, CD and FT-IR analysis showed that NS3 and NS5A contain both alpha-helical and beta-sheet structures and for NS5A, the proportion is almost equal. The production of NS3 and NS5A in milligram quantities will allow their characterization by biophysical and biochemical means that will help in designing new strategies to fight against HCV infection.

## Background

Hepatitis C Virus (HCV) was first identified in 1989 [1], however, research was initially delayed due to the difficulty to culture the virus efficiently *in vitro *[2]. HCV is a major public health threat worldwide that could lead to fibrosis, cirrhosis, hepatocellular carcinoma, liver failure and death of the infected person [3]. According to an estimate of World Health Organization, around 170 million people are infected with HCV, with an additional 3–4 million infected every year [4]. The risk of the prevalence of this disease is decreased with the advent of new blood screening tests. Yet, in developing countries, wide transmission still occur mainly by parenteral exposure to contaminated blood or blood products or by the illicit use of injectable drugs [5].

HCV belongs to genus hepacivirus of family Flaviviridae and is an enveloped positive stranded RNA virus [6]. It has a genome size of 9.6 kb and encodes a single polyprotein of 3000 amino acids which is further cleaved into 10 proteins by host signal peptidases and viral proteases [7,8]. Coding sequences for first 3 structural proteins reside in the 5′ region of HCV genome including the core (C), and two envelope glycoproteins (E1 and E2). The structural proteins play a major role in the encapsulation of the virus. C, E1 and E2 genes are followed by p7 gene whose product is considered to work as an ion channel and its maturation is catalyzed by endogenous peptidases. Coding regions of six non-structural proteins (NS2, NS3, NS4A, NS4B, NS5A and NS5B) are present at 3′ end of HCV genome. Non-structural proteins are mainly involved in process of replication and maturation of HCV [9].

In spite of the intensive research on HCV, it continues to pose a major threat to the developing world. The only treatment available for HCV is pegylated interferon alone or in combination with ribavirin for 24 to 48 weeks based on the genotype of virus. Response of genotype 1 to current treatment is 42 to 46% whereas genotypes 2/3 responds to 76 to 80% [10]. This antiviral therapy is expensive, has considerable side effects and in some cases is not effective beyond the period of treatment. A number of DAAs are in clinical phase 1, 2 and 3 trials that are designed against specific protein targets (NS3, NS5A and NS5B) which are essential to the life cycle of the virus [11-13]. Two DAAs Telaprevir and Boceprevir have been approved by FDA in 2011. These DAAs are used in combination therapy with interferon and ribavirin to combat infections caused by HCV genotype 1a [14]. NS3 and NS5B are considered to be the main targets of this regime because they can be directly used for inhibitors screening due to their enzymatic properties [15]. NS3 consists of 631 amino acids (aa), out of which N-terminal 180 aa form a protease domain while remaining constitutes helicase/ATPase part of the protein. The protease domain plays an important role in the processing of HCV polyprotein using the NS4A as a cofactor [16]. NS4A not only plays an important role in the activation of NS3 protease, it also anchors NS3 to membrane of endoplasmic reticulum during process of replication [17]. Helicase domain of NS3 activates the separation of double stranded RNA using ATP as an energy source [18]. Moreover, NS3 also interferes the host immune system by targeting the host proteins such as TRIF and MAVS that act as signaling adapter for toll like receptor 3 and mitochondrial antiviral signaling protein, respectively [16]. Although Protease and helicase domains of NS3 can work independently, cofactor NS4A is required for efficient functioning of NS3 protease [19].

NS5A performs critical role in the virus persistence and pathogenesis. Its interaction with a large number of host and viral proteins during replication and assembly of virus, makes this protein an important antiviral drug target [20,21]. NS5A also has significant role in the process of lipid accumulation that finally leads to steatosis [22,23]. Work on the discovery of NS5A′ inhibitors is continuing and so far only one inhibitor “Daclatasvir” is available that is in phase 3 clinical trials [13,24]. Daclatasvir is effective for treatment of infection caused by HCV genotype 1a. NS5A consists of 3 domains; domain I and domain II are essential for viral replication whereas domain III plays a key role in virion assembly [25,26]. The crystal structure of domain 1 has been solved, suggesting that NS5A forms a dimer that contains a groove for binding RNA at the interface between the monomers [27].

The prevalence of HCV in Pakistan/South Asia is on the rise, currently at approximately 10% [28]. The most prevalent genotype in this region is 3a [29]. Inhibitors such as Telaprevir, Boceprevir and TMC435 targeting NS3 are only effective against HCV genotype 1. MK-5172, another inhibitor of NS3, which is supposed to be effective against all genotypes, is still in clinical trials [30]. Research is progressing to exploit the potential of NS5A as an antiviral target [31].

First step towards designing effective inhibitors is to obtain sufficient quantities of the target protein. Such quantities will enable extensive functional and structural analyses required for the rational design of inhibitors. To our knowledge there is no systematic study has been performed to over-express and purify NS3 and NS5A of HCV genotype 3a. Considering the importance of NS3 and NS5A as antiviral drug targets, we have successfully devised over-expression and purification methods to produce the indicated proteins in quantities sufficient for functional and structural studies with and without inhibitors. In addition, we have functionally and structurally characterized HCV NS3 and NS5A proteins from genotype 3a.

## Results and discussion

**Figure 1:**
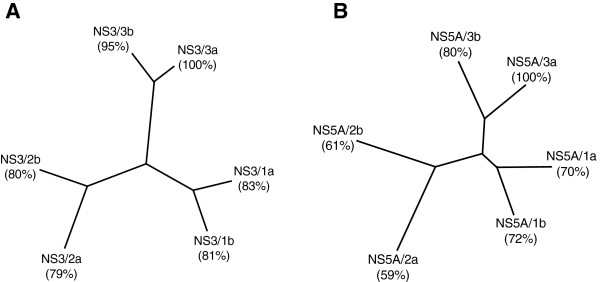
**Percentage sequence identities between NS3 [A] and NS5A [B] proteins of various genotypes of hepatitis C virus.** The phylogenetic trees and percent identity were derived from alignments of amino acid sequences of NS3 and NS5A proteins using the program ClustalX, version 1.83. Percent identity of NS3/3a and NS5A/3a to proteins of other genotypes is indicated. The tree was drawn using the TreeView program. **[A]** The phylogenetic tree includes: NS3/3a (NCBI GenBank acc. no. AFJ79449), NS3/3b (BAA08372; Amino acids 1035–1665), NS3/2a (BAB32872.1; Amino acid 1031–1661), NS3/2b (BAB08107; Amino acid 1031–1661)), NS3/1a (AAB66324; Amino acid 1027–1657), NS3/1b(BAC54896; Amino acid 1027–1657). **[B]** The phylogenetic tree includes: NS5A/3a (AFJ79451), NS5A/3b (BAA08372; Amino acid 1981–2432), NS5A/2b (AAF59945; Amino acid 1977–2442), NS5A/2a (AAF01178; Amino acid 1977–2442), NS5A/1a (AAB66324; Amino acid 1973–2420), NS5A/1b (AAC15725; Amino acid 1973–2419).

Infections caused by HCV pose major threats to human health. Recently launched ‘NS3’ inhibitors (Telaprevir, Boceprevir and TMC435) for treatment of HCV genotype are found to be ineffective against HCV patients infected with genotype 3a, which is prevalent in several developing countries [32]. This might be due to amino acid sequence variability among NS3 proteins from different genotypes (Figure 1A). In addition to NS3 as an antiviral drug target, significant efforts are now being made to exploit the potential of NS5A [33]. Compared to NS3 sequence variability, the sequence of NS5A is even more variable (30-40%) among different HCV genotypes (Figure 1B). This fact highlights the need to synthesize and characterize these target protein from different genotypes to combat HCV infection worldwide [34]. As a step forward, we have isolated HCV genotype 3 strain from blood sample of a Pakistani patient that was used for synthesis of viral cDNA. The nucleotide sequences coding for NS3 and NS5A proteins were amplified using the isolated cDNA as a template. Nucleotide sequences of NS3 and NS5A were deposited in GenBank NCBI under accession numbers JQ676838 and JQ676840, respectively.

### Strategy for the generation of NS3, NS5A and NS5A-T expression constructs

**Figure 2:**
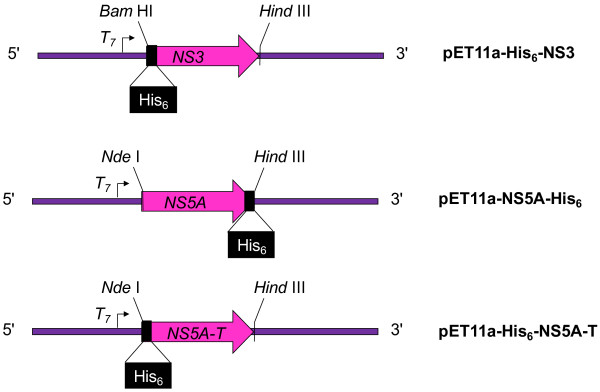
**Strategy and graphical representation of pET11a-based expression vectors employed in this study, illustrating the position encoded tag sequences, key restriction sites, T**_**7 **_**promoter and NS3, NS5A and NS5A-T (truncated sequence).**

Nucleotide sequences of NS3, NS5A (Full length; nucleotides coding for 1–452 amino acids) and NS5A-T (truncated; 32–452 amino acids) along with coding sequence for His_6_ at the 5′, 3′ and 5′ ends, respectively, were cloned into pET11a vector. Resultant vectors were named as pET11a-His_6_-NS3, pET11a-NS5A-His_6_ and pET11a-NS5A-T-His_6_ and overall strategy is shown in Figure 2. The Integrity of constructs was confirmed by DNA sequencing.

### Expression of NS3, NS5A and NS5A-T

Initial expression of His_6_-NS3, NS5A-His_6_ and His_6_-NS5A-T was conducted at 37°C using 1 mM IPTG concentration in *E. coli* strain BL21(DE3) as described in Methods section. Truncated version of NS5A-T (32–452 amino acids) was also tested to evaluate the effect of N-terminal domain of 31-amino acids (which contains an amphipathic helix that serves as a membrane anchor) on protein expression [35,36]. Significant expression levels of His_6_-NS3 and His_6_-NS5A-T were obtained whereas NS5A-His_6_ was expressed to low level as detected by Coomassie blue staining of SDS-polyacrylamide gel (data not shown). Therefore, further studies were conducted using truncated version of NS5A.

**Figure 3:**
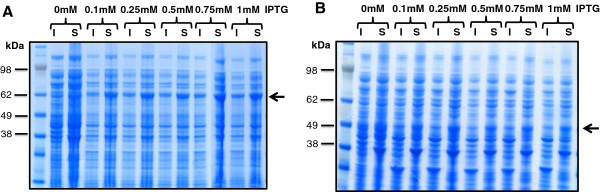
**Expression of His**_**6**_**-NS3 [A] and NS5A-T-His**_**6 **_**[B] in *E. coli *strain BL21(DE3) and isopropyl-β-D-thiogalactoside (IPTG).** ‘I’ and ‘S’ represent insoluble (inclusion body) and soluble fraction of lysate obtained from cultures harbouring pET11a- His_6_-NS3 **[A]** or pET11a- NS5A-T-His_6_**[B]** induced with indicated concentration of IPTG. The arrows indicate the mobility of monomeric His_6_-NS3 **[A]** and NS5A-T-His_6_**[B]**. The lane on the left shows marker proteins of known molecular mass.

To enhance the expression level and solubility of His_6_-NS3 and His_6_-NS5A-T/HCV genotype 3a, concentration of inducer ‘IPTG’ was optimized [37]. Previous studies report that expression of NS3/HCV genotype 1a/2b/3a usually obtained at 1 mM IPTG or 0.002% L-arabinose using pET or pBAD vector systems [38-40] whereas NS5A/HCV genotype 1a/1b is produced using 0.2-1 mM IPTG [21,41-43]. Various IPTG concentrations ranging from 0.1-1 mM as detailed in Methods section were employed for expression in this study. For both proteins, expression yielded bands migrating at ~ 68.3 and ~46.5 kDa for His_6_-NS3 and His_6_-NS5A-T, respectively (Figure 3). A significant proportion of proteins expressed as soluble at all IPTG concentrations. Overall, little variation in expression of both proteins was observed at IPTG ranging from 0.1 to 1 mM. For further expression studies, 1 and 0.5 mM IPTG concentrations were selected for His_6_-NS3 and His_6_-NS5A-T/HCV genotype 3a.

### Temperature optimization and purification of NS3 and NS5A-T

**Figure 4:**
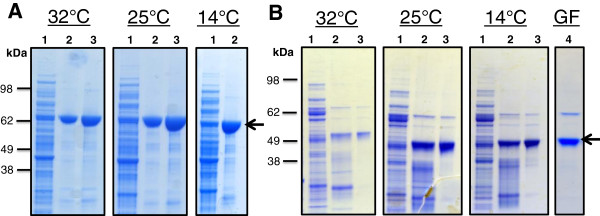
**Optimization of expression of His**_**6**_**-NS3 [A] and NS5A-T-His**_**6 **_**[B] in *E. coli *strain BL21(DE3) at various temperatures and purification by Nickel affinity chromatography following gel filtration in case NS5A-T-His**_**6 **_**[B].** SDS-polyacrylamide gels stained with Commassie brilliant blue, showed wash fraction (lane 1), purified protein fractions eluted from Ni-NTA column using 250 mM imidazole (lane 2&3) and purified fraction of NS5A-T-His_6_ by gel filtration as described in Methods section. The mobilities of marker proteins of known molecular mass are shown on the left.

**Figure 5:**
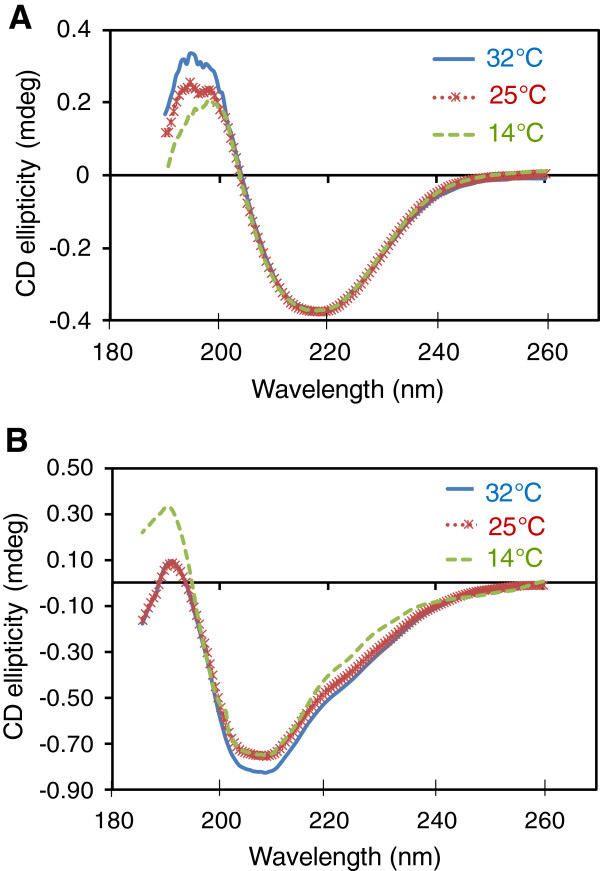
**Characterization of His**_**6**_**-NS3 [A] and NS5A-T-His**_**6 **_**[B] over-expressed at 32°C, 25°C and 14°C by circular dichroism spectroscopy.** CD experiments were performed as described in Methods.

Previous studies report expression of NS3 and NS5A from different HCV genotypes using a range of temperatures. For example, expression of NS3/HCV genotype 1a/2b/3a has been achieved at 30°C and 22°C [38-40], and NS5A has been expressed at room temperature in most of studies [21,41,43,44]. In this study, we systematically investigated the affect of temperature (32°C, 25°C and 14°C) on expression and purification yield of His_6_-NS3 and His_6_-NS5A-T/HCV genotype 3a. Both proteins can be expressed at all tested temperatures and purified by single step Ni-NTA affinity chromatography to ~ 95% purity as judged by Coomassie blue staining (Figure 4). His_6_-NS5A-T is further polished by gel filtration to produce homogenous and mono-disperse protein (Figure 4B, lane 4). Higher purification yields 4.0 and 1.0 mg per liter culture volume were obtained at both 25°C, 14°C and 25°C for His_6_-NS3 and His_6_-NS5A-T, respectively (Table 1). Optimized expression conditions yielded four fold purified His_6_-NS3 than achieved earlier [40] whereas His_6_-NS5A-T yield was somewhat similar as reported in earlier studies for NS5A or NS5A-T/HCV genotype 1 [41,44]. Higher purification yield of NS3/3a might be due to its sequence variability from NS3 sequences of other genotypes (Figure 1A). Identity of purified NS3 and NS5A was confirmed by peptide mass finger printing based mass-spectrometry method. CD analyses of the purified His_6_-NS3 and His_6_-NS5A-T produced at different temperatures (32°C, 25°C and 14°C) revealed that both proteins are folded polypeptides (Figure 5).

**Table 1:** Purification yield of NS3 and NS5A-T

**Protein**	**32°C**	**25°C**	**14°C**	**Reported**
**Temperature**				
**NS3 (mg/L)**^**1**^	2.5	4.0	4.0	0.3 [38], 0.92 [40], 0.45 [39]
**NS5A-T (mg/L)**^**1**^	0.32	1.0	0.8	1.0 [41]

^1^The yield of purified proteins were measured mg/liter of culture (expression medium).

### Activity measurements of NS3 produced at different temperatures

**Figure 6:**
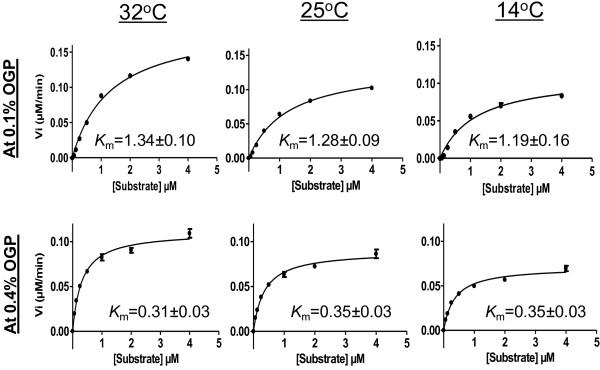
**Concentration dependence of Ac-Asp-Glu-Asp(EDANS)-Glu-Glu-Abu-ψ-[COO]-Ala-Ser-Lys(DABCYL)-NH2 [substrate] cleavage by NS3 produced at various temperatures.** Substrate-cleavage reactions were performed using 0.1 and 0.4% OPG as described in Methods. Data points shown were fitted to the Michaelis-Menten equation by non-linear regression.

Activity of purified His_6_-NS3 produced at 32°C, 25°C and 14°C was measured using a FRET based assay as described in Methods section. His_6_-NS3 expressed at different temperatures, exhibit similar *K*_m_ value when measured at single detergent concentration (0.1 or 0.4% n-Octyl-β-D-glucopyranoside; OGP) (Figure 6). Using assay buffer containing 0.1% OGP, higher *K*_m_ value (~1.3 μM) for His_6_-NS3 was obtained than at 0.4% OGP (~0.3 μM). *K*_m_ value in the range of 1.3 μM has been determined before for NS3/HCV genotype 1a [38]. Maximum turnover number (*K*_cat_) and specificity constant (*K*_cat_/*K*_m_) were found to be higher for the enzyme expressed at 32°C at both OGP concentration used in assay buffer (Table 2). However, taking into account the purification yield of His_6_-NS3 at various temperatures, the expression at 25°C seems to be the best to get the largest amount of enzyme, with catalytic efficiency sufficient for conducting assays for intensive search of inhibitors (Table 2). Relatively higher specificity constant (*K*_cat_/*K*_m_) for His_6_-NS3, expressed at 32°C might be due to local perturbation at around active site of the enzyme which could be the subject of future investigations.

**Table 2:** Catalytic efficiency of NS3 produced at various temperatures

**Protein**	**Temperature**	***K***_**cat **_**(s**^**-1**^**)**	***K***_**cat**_**/*K***_**m**_	***K***_**cat**_**/*K***_**m **_**(μM**^**-1**^ **· s**^**-1**^**)**
			**(μM**^**-1**^ **· s**^**-1**^**)**	**× Purification yield (mg/L**^**1**^**)**
**NS3 (0.1% OPG)**	32°C	3.17 ± 0.10	2.37	5.93
	25°C	2.27 ± 0.07	1.77	7.04
	14°C	1.836 ± 0.11	1.55	6.20
**NS3 (0.4% OPG)**	32°C	1.840 ± 0.05	5.93	14.82
	25°C	1.485 ± 0.04	4.22	16.88
	14°C	1.176 ± 0.03	3.28	13.12

^1^The yield of purified proteins were measured milligram per liter of culture (Expression medium).

Although similar *K*_m_ values were obtained for His_6_-NS3, expressed at various temperatures, *K*_cat_/*K*_m_ constants were different to some extent. To investigate, whether these differences relate to changes in overall structural features of the protein, CD analysis was performed. The CD spectra of His_6_-NS3 proteins expressed at different temperatures are almost identical in the region between 200 – 240 nm that reflects the secondary structure propensities in proteins (Figure 5A) [45]. Thus, it is likely that the overall structural features in His_6_-NS3 expressed at various temperatures remain intact and the cleavage efficiency of the protein does not relate to any global structural changes.

### Structural analysis of NS3 and NS5A-T

**Figure 7:**
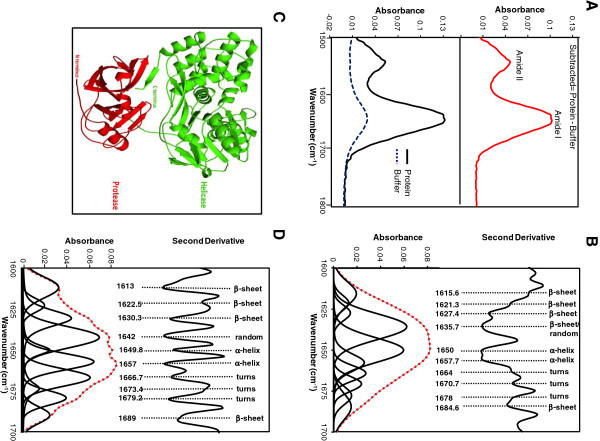
**Analysis of His**_**6**_**-NS3 [A,B,C] and NS5A-T-His**_**6 **_**[D] secondary structure by FT-IR and homology modeling. [A]** Lower panel: FT-IR spectra of hydrated film of 10 mM phosphate buffer pH 7.4 and His_6_-NS3 protein. Upper panel: Final protein spectrum obtained by subtracting the buffer spectrum. **[B]** Second derivative spectrum of His_6_-NS3 and amide I region of the FTIR spectrum of a hydrated film of His_6_-NS3 (thick grey line), and bands obtained by deconvolution. The dotted red line shows the curve fitted using the component bands. **[C]** Homology model of NS3/3a, built using the x-ray structure of NS3/1a as a template (PDB id: 3o8b). The protease and helicase domains are shown in red and green respectively. **[D]** Second derivative spectrum of NS5A-T-His_6_ and amide I region of the FT-IR spectrum of a hydrated film of NS5A-T-His_6_ (thick grey line), and bands obtained by deconvolution. The dotted red line shows the curve fitted using the component bands. The peak position of the spectral components along with their assignment is shown in the figure.

There are no 3 dimensional structures available for HCV NS3 genotype 3a or the full length NS5A of any genotype in protein data bank. Thus, to estimate secondary structure of NS3 and NS5A-T we used data from CD and FT-IR spectroscopy which indicate that both proteins fold into mixed secondary structure components (Figures 5A and B; 7B and D). To provide quantitative estimate of the alpha-helical and beta-sheet content of NS3, the measured amide I region of the spectrum (Figure 7A; Upper panel) could be reproduced by a fit of eight components, dominated by those indicative of alpha-helix and beta-sheet [46-48] (Figure 7B). Quantitative analysis of the spectrum predicted the presence of ~ 25% alpha-helix and ~ 34% beta-sheet structures (Table 3). Similar ratios were obtained from CD data and the homology model of HCV NS3 genotype 3a, built using the x-ray crystal structure of genotype 1a counterpart, as a template (Figure 7C; Table 3) [49,50].

**Table 3:** **Secondary structure analysis of His**_**6**_**-NS3 and NS5A-T-His**_**6 **_**by FT-IR, CD spectroscopy and homology modeling**

**Protein**	**Method**	**α-helix (%)**	**β-sheet (%)**
NS3	FT-IR^1^	25.7	34.6
	CD^2^	24.4	46
	CD^3^	11	37.6
	Homology model^4^	25	30
NS5A-T	FT-IR^1^	29	28
	CD^2^	9.1	32.7
	CD^3^	18	37

^1^Bands contributing (FT-IR spectra) to each type of secondary structure are indentified in Figure 7B and 7D respectively. Bands assignments for interpretation of spectra were made on the basis of previous measurements [48,58]. ^2^CD spectra shown in Figure 5 were analyzed using protein concentration independent method at web based server http://perry.freeshell.org/raussens.html [52]. ^3^CD spectra were analyzed using DichroWeb server at http://dichroweb.cryst.bbk.ac.uk/html/home.shtml [53,54] and program CONTIN/reference set 3 [55]. ^4^Secondary structure are estimated from the homology model of NS3/3a, built using the x-ray structure of NS3/1a (PDB ID: 3o8b) as a template [49].

Secondary structures for NS5A, quantified by FT-IR were found to be around ~ 29% alpha-helix and beta-sheet each whereas CD analysis revealed ~18% alpha-helix and ~32% beta-sheet (Table 3). This discrepancy particularly in case of alpha-helix contents might be due to different algorithms used for analysis of CD and FT-IR data.

## Conclusions

We have successfully optimized conditions for the recombinant expression and purification of the NS3 and NS5A proteins from HCV genotype 3a. Yield of the purified NS3 from soluble fraction of *E. coli* cells lysate is 4 mg per liter culture volume, which is the highest amount, obtained for this genotype to the best of our knowledge. CD studies show that difference in activities of NS3 produced at different temperatures is not due to changes in the overall structural features of the expressed proteins. CD and FT-IR spectra suggest that both proteins are folded into a mix of alpha-helix and beta-sheet secondary structure. Procedure described here to produce milligram quantities of purified NS3 and NS5A is expected to allow rigorous biochemical and biophysical characterization, and screening of inhibitors to combat HCV infection, in particular of genotype 3.

## Methods

### Extraction of RNA and cDNA synthesis

Blood samples were collected from different hospitals, already involved in work of HCV diagnostic services (Punjab Institute of Nuclear Medicine, Faisalabad Pakistan and Liver Center, District Head Quarter Hospital, Faisalabad, Pakistan). Informed consent was taken from every donor and approval of institutional ethical committee was obtained to conduct such studies. Plasma was separated from blood samples by centrifuging them and stored at -20°C until RNA extraction. Total RNA was extracted from these samples infected with HCV genotype 3a using the Viral RNA extraction kit (MACHERY-NAGEL, Germany) according to manufacturer’s instruction. 140 μl of plasma sample was used to extract RNA and finally eluted using the 60 μl elution buffer supplied with kit. RNA was quantified using the nanodrop (Thermo Scientific) and stored at -80°C if needed. cDNA was synthesized from the extracted RNA using the primers for NS3 and NS5A (NS3-cDNA and NS5A-cDNA, Table 4) with RevertAid™ H Minus First strand cDNA synthesis kit (Fermentas, Germany) according to manufacturer’s recommendations.

**Table 4:** Oligonucleotides used in this study

**Name**	**Sequence**
NS3-F	5*'*-TATA*GGATCC*ATGCACCATCACCATCATCACGCCCCGATCACAGCATAC-3*'*
	*Bam*HI
NS3-R	5*'*-GAGC*AAGCTT*TTAGGTGGTTACTTCCAGATCAG-3*'*
	*Hind* III
NS5A-F	5*'*-GGAATTC*CATATG*AGCGGTGATTGGCTGCGTA-3*'*
	*Nde*I
NS5A-R	5*'*-TCA*AAGCTT*TTAGTGGTGATGGTGATGATGGCAGCAGACCACGCTCTGCT-3*'*
	*HindIII*
NS5A-T-F	5*'*-GGAATTC*CATATG*CACCATCACCACCATCACCCTGGGCTGCCCTTTATTTCCT-3*'*
	*Nde*I
NS5A-T-R	5*'*-TCA*AAGCTT*TTAGCAGCAGACCACGCTCTGCT-3*'*
	*Hind* III
NS3-cDNA	5*'*-ACCCCTCCAAGCAACACCCA-3*'*
NS5A-cDNA	5*'*-ATGTTGCTGCCCATCTCTTG-3*'*

Nucleotides shown as italicized represent the respective restriction sites used for cloning purposes whereas underlined nucleotides sequence encode for oligohistidine tag, incorporated into genes of interest for downstream purification processes.

### PCR amplification and construction of expression constructs

Nucleotide sequences of NS3A and NS5A of a number of isolates of HCV genotype 3a were retrieved from the European Hepatitis C Virus database (http://euhcvdb.ibcp.fr/euHCVdb/) and primers were designed from the consensus sequences of NS3, NS5A and NS5A-T (lacking N-terminal 31 amino acids), using vector NTI software (Invitrogen). Nucleotide coding regions for NS3, NS5A and NS5A-T were amplified from respective cDNA (see section “Extraction of RNA and cDNA synthesis”) through gradient polymerase chain reaction (PCR) using NS3-F/R, NS5A-F/R and NS5A-T-F/R, respectively (Table 4) and relevant restriction sites/nucleotides encoding for 6 × His tag were also incorporated. Each reaction mixture of 50 μl contains 5 μl of cDNA, 0.2 mM dNTPs, 2 mM MgSO_4_, 0.5 μM of each forward and reverse primer, 1.25 U of *Pfu* DNA Polymerase and 1X *Pfu* buffer (200 mM Tris–HCl pH 8.8, 100 mM (NH_4_)_2_SO_4_, 100 mM KCl, 1% Triton X-100, 1 mg/mL BSA (Fermentas, Germany).

The procedures for cloning were based on standard methods [51] and protocols recommended by the suppliers. Purified PCR products corresponding to NS3, NS5A and NS5A-T were restricted using the restriction enzymes *Bam*HI/*Hind*III, *Nde*I/*Hind*III and *Nde*I/*Hind*III, respectively and ligated into pET11a vector that was restricted using same restriction enzymes. The resulting constructs were named as pET11a-His_6_-NS3, pET11a-NS5A-His_6_ and pET11a-NS5A-T-His_6_. The identity of plasmids was confirmed by nucleotide sequencing. All genetic manipulation was performed in *Escherichia coli* strain XL1-Blue (Stratagene, USA). Nucleotide sequences of NS3 and NS5A were deposited in GenBank NCBI under accession numbers JQ676838 and JQ676840, respectively.

### Expression of NS3, NS5A and NS5A-T in *E. coli*

For initial expression trials, pET11a-His_6_-NS3, pET11a-NS5A-His_6_ and pET11a-NS5A-T-His_6_ were transformed into the *E. coli* strain BL21(DE3) cells and were grown at 37°C in a 50 mL of LB media supplemented with 100 μg/mL of ampicillin until OD_600_ reaches 0.5-0.6. The cells were then induced with 1 mM isopropyl β-D-1-thiogalactopyranoside (IPTG) and were grown for additional three hours. *E. coli* cells expressing His_6_-NS3, NS5A-His_6_ and His_6_-NS5A-T were harvested by centrifugation at 5000 rpm using the Beckman centrifuge and resuspended in lysis buffers, 25 mM HEPES [4-(2-hydroxyethyl)-1-piperazineethanesulfonic Acid] pH 7.6 containing 20% glycerol, 0.5 M NaCl, 2.5 mM β-mercaptoethanol, 0.2% n-dodecyl-β-D-maltopyranoside and 100 mM Tris–HCl pH 8.0 containing 0.5% triton; 200 mM NaCl; 10 mM β-Mercaptoethanol and 10% glycerol, respectively. Before sonication, in case of NS5A cell resuspensions were supplemented with few μg of DNase, RNase and 1-tablet of EDTA-free protease cocktail inhibitor (Roche, Germany). Following disruption of cells by sonication, soluble fraction was separated from insoluble portion by spinning at 12000 × *g* for 30 min. The pellet representing the insoluble fraction was dissolved in respective lysis buffer and equal volume of both soluble and insoluble fractions were analyzed on SDS-polyacrylamide gel.

To optimize the inducer concentration for expression of His_6_-NS3 and His_6_-NS5A-T, various quantities of IPTG 0.1 mM, 0.25 mM, 0.5 mM, 0.75 mM and 1 mM IPTG were used in expression experiments and analyses were performed as described before. To enhance solubility and yield of His_6_-NS3 and His_6_-NS5A-T, following induction of cultures using 1 mM and 0.5 mM IPTG respectively, optimization of temperature was carried out at 32°C, 25°C and 14°C. For 32°C, 25°C and 14°C post-induction time was kept 4 and 12 hours respectively. Expressed proteins were analyzed by SDS-PAGE.

### Purification of NS3

To estimate yield of purified His_6_-NS3 at various post-induction temperatures 32°C, 25°C and 14°C, 1.5 L expression culture was set up at each temperature. Before IPTG induction, culture media was supplemented with 100 μM of ZnCl_2_.

For purification, the ÄKTA explorer platform (GE Healthcare) was used. All purification procedures were performed at 4°C.The pellet was resuspended in 25 mL lysis buffer (25 mM HEPES [4-(2-hydroxyethyl)-1-piperazineethanesulfonic Acid] pH 7.6 containing 20% glycerol, 0.5 M NaCl, 2.5 mM β-mercaptoethanol, 0.2% n-dodecyl-β-D-maltopyranoside. The cells were lysed using cell disrupter at 17 Kpsi (Constant Systmes, Ltd) and lysate was centrifuged for 50 min at 12000 × *g*. The pellet was discarded and supernatant was loaded on the chelating sepharose FastFlow gel charged with Nickel (GE healthcare) pre-equilibrated with lysis buffer. To remove non-specific loosely bound protein from Nickel-sepharose matrix, a step gradient with increasing concentration of imidazole (5 mM and 50 mM) in lysis buffer was used. His_6_-NS3 was then eluted using the lysis buffer except containing 250 mM imidazole.

The peak protein fractions were pooled and applied on PD-10 desalting columns (GE Healthcare) pre-equilibrated with buffer (25 mM HEPES pH 7.6 containing 20% glycerol, 0.2 M NaCl, 10 mM β-mercaptoethanol, 0.1% n-dodecyl-β-D-maltopyranoside) to remove the excess of imidazole. Concentration of purified His_6_-NS3 was measured using nanodrop and Pierce™ 660 nm protein assay (Thermo Scientific). The purified protein was stored at -80°C in small portions. Purity of the purified protein was judged by SDS-PAGE.

### Purification of NS5A-T

For purification of His_6_-NS5A-T, cultures were grown exactly as explained for His_6_-NS3 except protein expression was induced using 0.5 mM IPTG. Cells containing over-expressed His_6_-NS5A-T were lysed and processed as described before for His_6_-NS3. Soluble fraction from lysate was loaded on sepharose-Nickel column pre-equilibrated with lysis buffer (100 mM Tris–HCl pH 8.0 containing 0.1% triton, 200 mM NaCl, 10 mM β-Mercaptoethanol and 10% glycerol). The column was washed with 10 and 3 column volume lysis buffer containing 10 and 60 mM imidazole, respectively. The protein was eluted using lysis buffer containing 300 mM imidazole. Peak fractions were pooled and concentrated using 30 kDa cutoff Vivaspin column (Sartorius, Germany). The concentrated protein sample was then loaded onto superdex 200 gel filtration column pre-equilibrated with the buffer (25 mM HEPES pH 7.5 containing 150 mM NaCl, 2 mM DTT and 10% glycerol) and let the sample pass through the column with a flow rate of 0.5 mL/min. The fractions with high UV absorption were collected and analyzed by SDS-PAGE.

### Circular dichroism spectroscopy analysis of NS3 and NS5A-T

Structural analysis of His_6_-NS3 produced at 32°C, 25°C and 14°C were performed using CD spectroscopy. Measurements were carried out using J-810, Jascon spectroplolarimeter at 25°C with constant nitrogen flushing. Samples (300 μl) containing the purified His_6_-NS3 and His_6_-NS5A-T at a concentration of 0.02 mg/mL (in 10 mM potassium phosphate pH 7.4 buffer supplemented with 20% glycerol, 0.1% n-octyl-β-D-glycopyranosid) and 0.05 mg/mL (in 10 mM potassium phosphate pH 7.4 buffer) respectively, were added into a Hellma quartz cuvette of 1 mm path length and scanned between 185 and 260 nm, averaging at least 10 accumulations. Similarly, blank samples containing 10 mM phosphate buffer pH 7.4 supplemented with 20% glycerol, 0.1% n-octyl-β-D-glycopyranosid or 10 mM phosphate buffer pH 7.4 were scanned. Spectra were recorded at a speed of 1 nm/15 sec, sensitivity 50 mdeg, bandwidth 0.5 nm, resolution 1 nm and response time 15 sec.

CD data were analyzed using protein concentration independent method at web based server http://perry.freeshell.org/raussens.html [52] and DichroWeb server at http://dichroweb.cryst.bbk.ac.uk/html/home.shtml [53,54] and program CONTIN/reference set 3 [55].

### Activity measurements of NS3

The protease activity was measured with fluorescence resonance energy transfer assay (FRET) using the depsipeptide substrate Ac-Asp-Glu-Asp(EDANS)-Glu-Glu-Abu-ψ-[COO]-Ala-Ser-Lys(DABCYL)-NH2 (AnaSpec, San Jose, CA, USA). NS3 Protease cleave this substrate resulting in production of fluorescence which can be measured continuously on a fluorescence plate reader (Fluoroskan, Ascent Labsystems, Sweden) with excitation and emission wavelengths at 355 and 510 nm, respectively. The cofactor for NS3 protease activity is a synthetic peptide “KKGSVVIVGRIVLSGK” which is the central part of HCV NS4A. For activity measurement, purified His_6_-NS3 was diluted to 1 nM with the assay buffer (50 mM HEPES pH 7.5 containing 10 mM DTT, 40% glycerol and 0.1 or 0.4% n-octyl-β-D-glycopyranoside), pre-incubated for 10 min at 30°C with 25 μM NS4A-peptide (dissolved in dimethyl sulfoxide). The assay buffer with 0.1 or 0.4% OPG was used in order to see the effect of detergent concentration on activity of NS3. The reaction was started by adding 10 μl of depsipeptide substrate in 2 fold dilution with highest concentration of 4 μM in a microtitre plate. The last well in microtitre plate was used as control containing no substrate in it. Corrections for inner filter effect of substrate were also performed [56]. Kinetic properties of enzyme (*K*_m_, *K*_cat_ and *K*_cat_/*K*_m_) were measured by fitting the Michaelis-Menten equation by non-linear regression using the GraFit software (Erithacus Software, Staines, MX, UK).

### FT-IR analysis of NS3 and NS5A-T

For infrared spectroscopy, purified His_6_-NS3 and His_6_-NS5A-T in 10 mM potassium phosphate buffer pH 7.4 supplemented with 0.1% n-dodecyl-β-D-maltopyranoside and 10 mM potassium phosphate buffer pH 7.4, respectively, were concentrated to ~ 5–6 mg/mL. A small amount (~ 15 μl) of sample was dried on platinum ATR platform (diamond crystal) to form a hydrated film and then spectra obtained using Bruker FT-IR spectrometer as described [57]. Briefly FT-IR spectra from 4000 to 500 cm^-1^ were collected in absorbance mode at 1 nm resolution, 256 scans coaddition and Blackman-Harris-3-term apodization. Similarly, spectra was recorded for buffer alone and subtracted from protein spectra to eliminate bending vibration of H_2_O that gives absorption band at around 1645 cm^-1^ in the amide I region. Subtracted protein spectra were manipulated by adjusting the subtraction factor until a flat baseline was obtained in the region between 2000–1770 cm^-1^. Where necessary, subtraction of residual vapour absorption was also performed.

For secondary structure analysis, second derivative of spectra corresponding to amide I region (1700–1600 cm^-1^) was obtained using the Origin software version 7.0 (OriginLab Corporation, USA) following Savitzky-Golay method using the parameters 3^rd^ grade polynomial, point to left/right : 2 and five smoothing points. Before performing second derivative analyses, spectra were smoothened using 2 adjacent points. Decomposition of the spectra into individual bands in the amide I region was performed by non-linear peak fitting using Galactic PeakSolve™ software (version 1.05). Band assignments for interpretation of spectra were done on the basis of previous measurements [48,58].

### Homology modeling

The 3D structure of HCV-3a NS3 protein was predicted through homology modeling, using the SWISS-MODEL Workspace [50,59]. 629 amino acids (3–631) were modeled using the x-ray structure of HCV-1a NS3 protein as a template (PDB ID: 3o8b, sequence identity 83%). The homology model was further idealized using the CCP4 program suite [60,61]. The final model was validated using the NIH MBI Laboratory for Structural Genomics and Proteomics Structural Analysis and Verification Server that allow use of five programs (Procheck, What_Check, ERRAT,Verify_3D, and Prove) to analyze the stereochemical parameters and quality of the model [62-66].

## Competing interests

The authors declare that they have no competing interests.

## Authors’ contributions

MIA performed cloning, expression, purification and kinetic work, and assisted in writing the manuscript. MI performed CD and IR analysis, and drafted the manuscript. MSY built the homology model and helped in drafting of the manuscript. MR conceived the idea of study, designed, coordinated the research and written the manuscript. All authors read and approved the final manuscript.
